# Performance Evaluation of Reinforced Concrete Beams with Corroded Rebar Strengthened by Carbon Fiber-Reinforced Polymer

**DOI:** 10.3390/polym17081021

**Published:** 2025-04-10

**Authors:** Sangwoo Kim, Wonchang Choi, Jinsup Kim

**Affiliations:** 1 Department of Civil Engineering, Gyeongsang National University, Jinju 52828, Republic of Korea; kimsangwoo92@gnu.ac.kr; 2 Department of Architectural Engineering, Gachon University, Seongnam 13120, Republic of Korea

**Keywords:** RC beam, corrosion, resilience, CFRP reinforcement, energy dissipation capacity

## Abstract

The inefficiency of unreinforced concrete beams as flexural members poses a challenge because concrete’s tensile strength is significantly lower than its compressive strength. In response to this challenge, reinforcement bars are commonly employed near the tension zone of reinforced concrete (RC) beams. Nonetheless, structures constructed with RC face challenges such as reduced live load capacity, concrete deterioration, and the corrosion of reinforcement bars over time. To address this, ongoing research is exploring maintenance and retrofitting techniques using high-strength, lightweight fiber-reinforced polymer (FRP) composite materials such as carbon fiber-reinforced polymer (CFRP) and glass fiber-reinforced polymer (GFRP). In this study, the flexural performance of corroded RC beams was enhanced through retrofitting with CFRP plates and sheets. The corroded RC beams were fabricated using an applied-current method with a 5% NaCl solution to induce a 10% target corrosion level under controlled laboratory conditions. Flexural tests were conducted to evaluate the structural performance, failure modes, load–displacement relationships, and energy dissipation capacities. The results showed that CFRP reinforcement mitigates the adverse effects of corrosion-induced reduction in rebar cross-sectional areas, leading to increased stiffness and improved load-carrying capacity. In particular, CFRP reinforcement increased the yield load by up to 36.5% and the peak load by up to 90% in corroded specimens. The accumulated energy dissipation capacity also increased by 92%. These enhancements are attributed to the effective load-sharing behavior between the corroded rebar and the CFRP reinforcement.

## 1. Introduction

With ongoing advancements in materials and construction techniques, reinforced concrete (RC) has become the most widely used construction material worldwide [1,2,3,4]. However, RC structures are still susceptible to various deterioration mechanisms due to aging, environmental exposure, and sustained loading, particularly steel reinforcement corrosion, which significantly compromises structural capacity and service life [5,6,7,8,9,10,11]. Hence, to counteract the decrease in the load-carrying capacity of concrete structures, research is underway on maintenance and retrofitting using high-strength, lightweight fiber-reinforced polymer (FRP) composite materials such as carbon fiber-reinforced polymer (CFRP) and glass fiber-reinforced polymer (GFRP) [12,13,14,15,16,17,18,19].

Recent studies have investigated the flexural performance and failure mechanisms of corroded concrete members strengthened with FRP materials [20]. These works highlight the relationship between corrosion level and structural degradation and demonstrate that FRP reinforcement mitigates performance loss. In addition to external bonding methods, prestressed CFRP systems with end anchors have been reported as an efficient retrofitting approach [21,22]. In addition to FRP systems, alternative strengthening techniques such as textile-reinforced mortar (TRM) have emerged as promising solutions for RC members. TRM systems, which use inorganic mortar matrices instead of epoxy, provide better resistance to elevated temperatures and are more suitable for fire-prone environments. Recent research has demonstrated the effectiveness of TRM in strengthening RC elements under thermal and mechanical loads [23].

As problems such as the corrosion and decreased adhesion strength of reinforcement plates have emerged over time with the traditional method of reinforcing concrete structures using steel plates and adhesive bonding, the application of FRP as an alternative to steel plates for structural reinforcement has been tested. FRP composites offer superior non-corrosive properties and excellent strength, enhancing the durability of construction structures. Moreover, their lightweight nature facilitates easier transportation and installation, thereby reducing construction time and improving workability [24,25,26,27]. Leveraging these advantages, active research is being conducted on retrofitting methods employing FRP materials. However, it is important to note that FRP systems—especially those using epoxy resins—are sensitive to elevated temperatures. Exposure to temperatures above the glass transition temperature of the matrix can result in a significant loss of stiffness and strength. This thermal vulnerability limits their use in fire-prone environments unless proper insulation or protection is provided.

**Figure 1:**
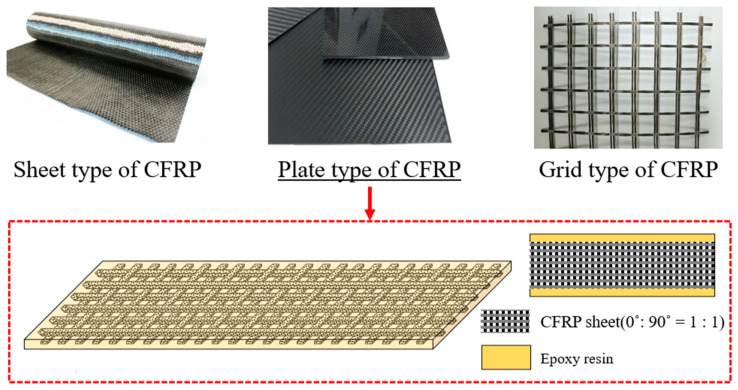
Types of carbon fiber-reinforced polymer (CFRP) reinforcement.: (top) sheet, plate, and grid types; (bottom) detailed schematic of the plate type CFRP. The underlined label and red arrow indicate that the schematic refers to the plate-type CFRP. The dashed box shows the layered structure of the CFRP plate and epoxy resin.

FRP reinforcement materials commonly include sheets, plates, and grid forms. As shown in Figure 1, plate-type CFRP is a product formed by impregnating high-strength CFRP sheets into epoxy resin. It demonstrates excellent tensile strength properties.

In this study, to enhance the flexural performance of corroded RC beams, two types of CFRP (CFRP plate and CFRP sheet) reinforcements were applied for retrofitting, and flexural experiments were conducted. Based on the experimental results, the failure modes, load history curves, and energy dissipation capacities due to corrosion and reinforcement were compared and analyzed. This study’s novelty lies in the combined use of CFRP plates and sheets for strengthening corroded RC beams under cyclic loading. Furthermore, it quantitatively evaluates not only the load-bearing capacity but also energy dissipation characteristics, offering new insights for seismic retrofitting of deteriorated RC structures.

## 2. Experimental Design and Reinforcement Method

### 2.1. Fabrication of Test Specimens

**Figure 2:**
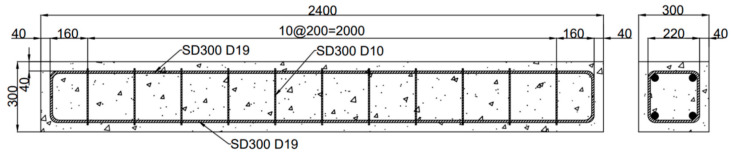
A schematic of the reinforced concrete (RC) beam (unit: mm). Arrows indicate measurement directions. The triangle and dot pattern represents the concrete section.

Four rectangular RC beams were designed for the test. The total length of each RC beam was 2400 mm, and the cross-section was 300 mm wide and 300 mm high. The thickness of the concrete cover was 40 mm. Figure 2 shows the geometry and reinforcement details of the RC beam. The tensile zone and compressive zone of the RC beam were equipped with two longitudinal steel bars with diameters of 19 mm each. Stirrups with diameters of 10 mm were arranged along the length of the beam. To avoid shear failure prior to flexural failure during the whole loading stage, the stirrup spacing in both the bending shear region and the pure bending region was set to 200 mm. Strain gauges were installed at the center of the two longitudinal steel bars positioned in the tensile zone of the RC beam to measure the deformation of the steel bars in accordance with the flexural deformation of the RC beams.

The experimental variables of this study are summarized in Table 1. RC1 represents the reference specimen, which is not reinforced and does not exhibit corrosion on the steel bars. RC2 indicates specimens with 10% corrosion of the steel bars. RC3 represents specimens reinforced with CFRP. RC4 indicates specimens where CFRP reinforcement was applied after 10% corrosion of the steel bars.

### 2.2. Material Properties of RC Beams

Ready-mixed concrete was used to cast the RC beams. Three cylindrical concrete specimens were used to measure the compressive strength of the concrete based on ISO 1920-4 [28], and the mechanical properties of steel reinforcement were obtained from the average of three coupon samples that were tested according to ASTM A370 [29]. Compressive tests were conducted 28 days after underwater curing, resulting in an average concrete compressive strength of approximately 24.5 MPa. The yield strength of the steel bar was 347 MPa, and the peak strength was 537 MPa.

### 2.3. Corrosion Experiment of RC Beams

**Figure 3:**
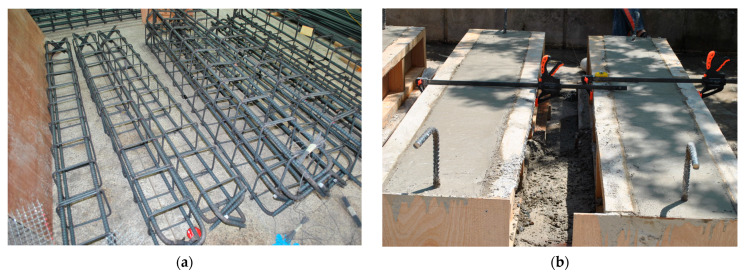

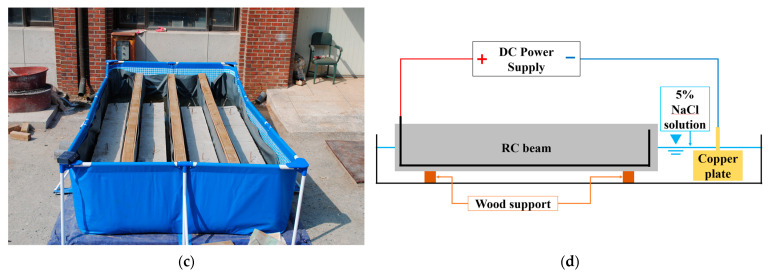
Specimen preparation process for RC beam testing. (**a**) Rebar assembly for RC beams. (**b**) Concrete casting and curing process. (**c**) Corrosion induction setup with multiple beams. (**d**) Schematic of accelerated corrosion system using 5% NaCl solution and DC power supply.

The applied-current corrosion method [30,31] is often used to quickly corrode the steel bars in RC structures. The specimen preparation process is illustrated in Figure 3. As shown in Figure 3a, longitudinal and transverse steel reinforcements were assembled according to the design specifications and prepared for placement in formwork. In Figure 3b, concrete was cast into wooden molds containing the rebar cages and cured under controlled conditions. After curing, selected RC beam specimens were submerged in a 5% NaCl solution for corrosion induction, as shown in Figure 3c. A copper plate was placed in the solution and connected to the negative terminal of a DC power supply, while the internal steel reinforcement was connected to the positive terminal. The detailed setup of the accelerated corrosion system is presented in Figure 3d.

A timer was used to control the corrosion duration. Corrosion rates were controlled over time, with a target corrosion rate set at 10%. After completing the corrosion process, the concrete cover was removed, and the corroded steel rebars were extracted. The corrosion rate was calculated by comparing the weight loss of the rebars before and after corrosion, using the following formula: Corrosion rate (%) = [(Initial weight − Corroded weight)/Initial weight] × 100

**Figure 4:**
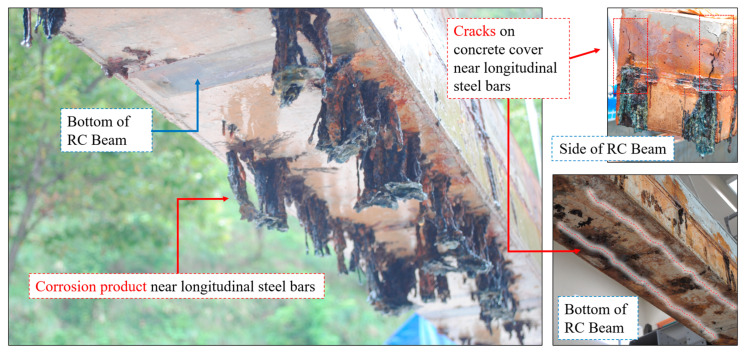
Photographs of corroded reinforced concrete (RC) beams and corrosion defects.

Although the applied-current corrosion method accelerates the corrosion process, it replicates key characteristics of natural corrosion, such as cracking due to rust expansion and degradation of the bond between concrete and steel. This method is widely accepted in research to simulate corrosion environments similar to those found in marine structures or de-icing salt exposure [7,31]. As shown in Figure 4, corrosion caused cracks to develop on the surface where the longitudinal steel bars are located at the bottom of the RC beam, and corrosion products can be observed seeping from the cracks.

### 2.4. Material Properties of CFRP Reinforcements

**Figure 5:**
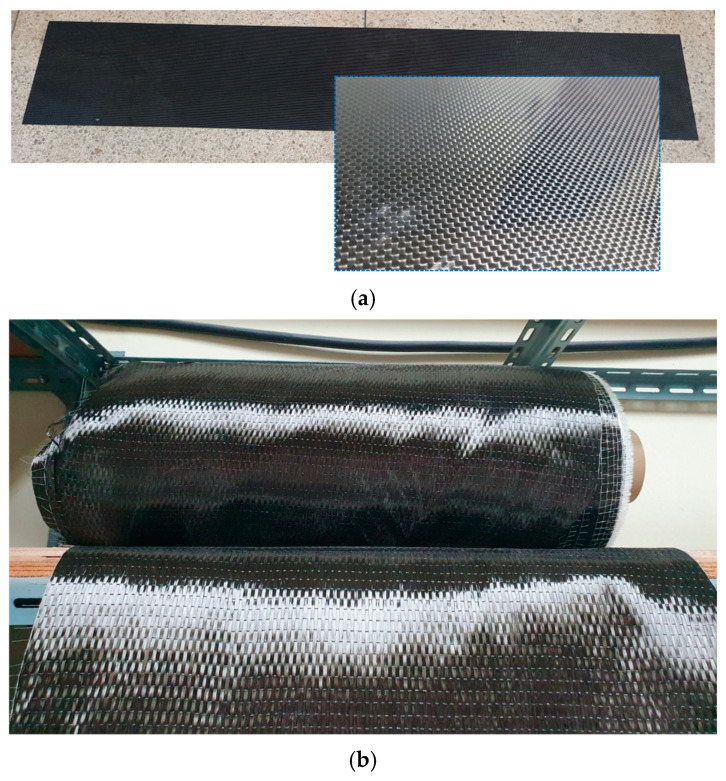
Photographs of carbon fiber-reinforced polymer (CFRP) reinforcements. (**a**) CFRP plate; (**b**) CFRP sheet.

**Figure 6:**
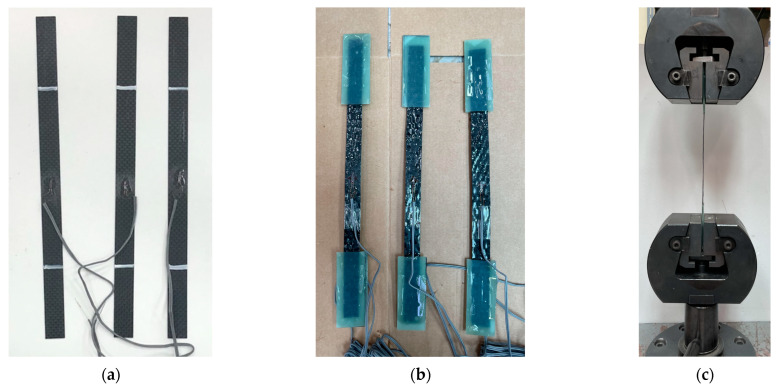
Tension test. (**a**) CFRP plate test coupons. (**b**) CFRP sheet test coupons. (**c**) Universal testing machine.

CFRP plates and CFRP sheets were used for the flexural reinforcement of the strengthened RC beams. The CFRP plate was formed by impregnating the CFRP sheet with epoxy resin and curing it into a rectangular shape. The shape of the CFRP plate is depicted in Figure 5a, and the CFRP sheet is shown in Figure 5b. The ultimate strength, ultimate strain, and elastic modulus of the CFRP plate and CFRP sheet were measured through coupon tensile tests (ASTM D 3039) [32]. Tension test coupons for the CFRP plate were fabricated, as shown in Figure 6a, and CFRP sheet test coupons were made from a CFRP sheet and an epoxy-binding agent (ASTM-D638) [33], as shown in Figure 6b. The unit weight and thickness of each CFRP plate coupon were 300 g/m^2^ and 0.5 mm, respectively. For the CFRP sheet test coupons, they were 300 g/m^2^ and 0.4 mm, respectively. Three coupons were prepared, as shown in Figure 6, and tested under tensile loading using a universal testing machine (UTM), as shown in Figure 6c.

All test results are summarized in Table 2. All values represent the mean results of three tested specimens. The average values for the tensile strength (ultimate strength), ultimate strain, and elastic modulus for the CFRP plate were 621.34 MPa, 0.024, and 25.89 GPa, respectively. From the CFRP sheet coupon tests, the tensile strength (ultimate strength), ultimate strain, and elastic modulus for the CFRP plate were 1367.72 MPa, 0.010, and 136.77 GPa, respectively. It is noted that the CFRP plate and sheet specimens tested in this study were fabricated using a wet lay-up process with hand-applied epoxy and ambient curing conditions. As such, the measured mechanical properties may be lower than those of commercially produced, autoclave-cured CFRP products typically used in structural engineering applications.

### 2.5. Strengthening Technique for RC Beams with CFRP Reinforcements

A CFRP plate was attached to the bottom part of the RC beam, and a CFRP sheet was affixed over the CFRP plate to prevent it from detaching under bending loads. This ensures that the CFRP plate can withstand bending until it reaches its breaking point.

**Figure 7:**
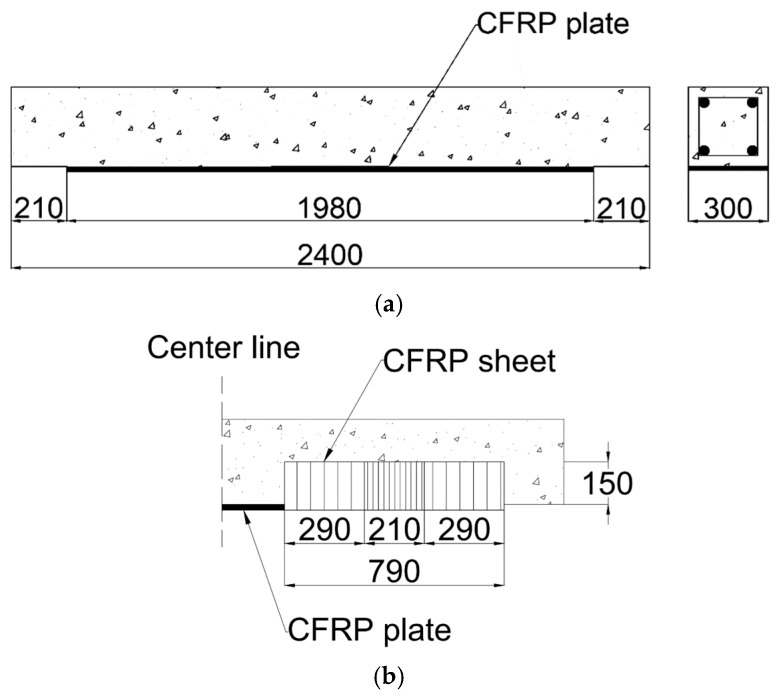

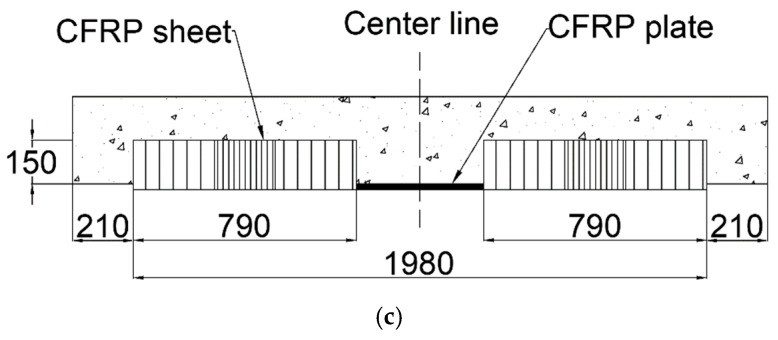
A diagram of the carbon fiber-reinforced polymer (CFRP) strengthening method for a reinforced concrete beam (unit: mm). (**a**) An RC beam with a CFRP plate; (**b**) an RC beam with a CFRP sheet; (**c**) an RC beam strengthened with a CFRP sheet and plate. The triangle symbol indicates the section line representing the concrete cross-section.

The size of the CFRP plate was manufactured to be 300 mm wide and 1980 mm long, and it was attached to the center of the underside of the RC beam, as shown in Figure 7a. To prevent the detachment of the CFRP plate according to the flexural capacity of the CFRP plate-reinforced RC beam and to demonstrate sufficient reinforcement performance, CFRP sheets were attached longitudinally to the ends of the CFRP plate, as shown in Figure 7b. The CFRP sheets were attached over an area measuring 790 mm in length and 600 mm in width, ensuring sufficient attachment length by positioning the CFRP sheet at the center of the beam’s side. Since the CFRP sheet was manufactured to be 500 mm wide, it was attached with an overlap length of 210 mm. The final reinforcement design details and dimensions are depicted in Figure 7c.

**Figure 8:**
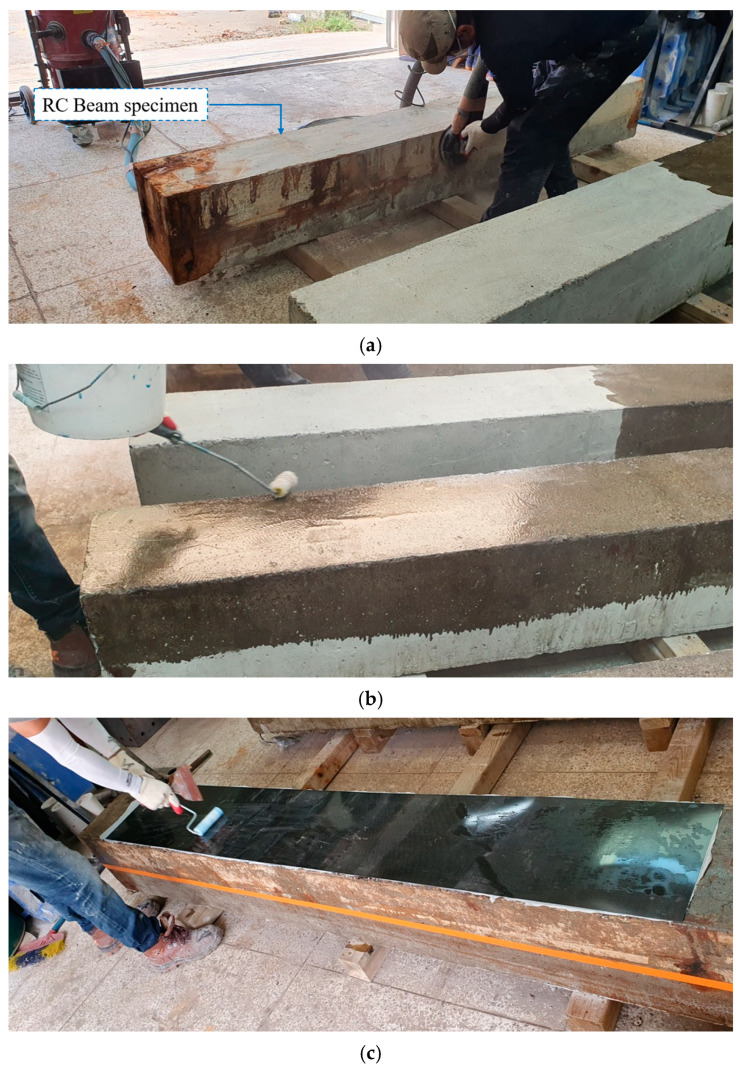

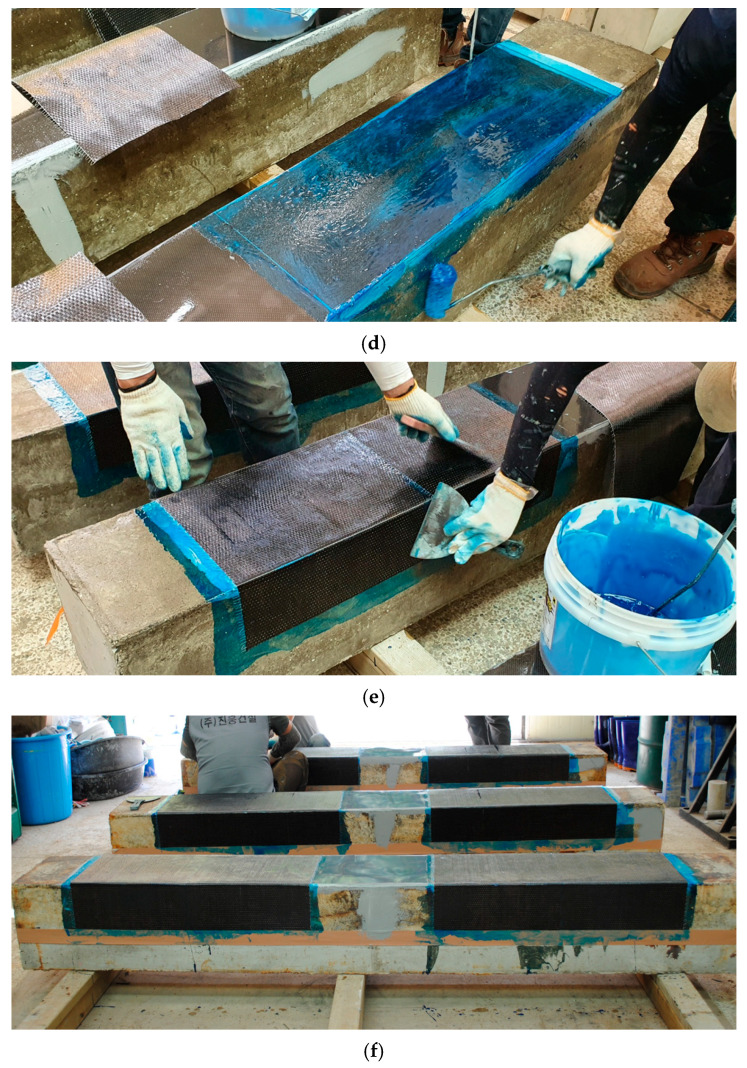
The carbon fiber-reinforced polymer (CFRP) installation procedure for strengthening reinforced concrete (RC) beams. (**a**) Roughening and cleaning the tension surface of the RC beam; (**b**) applying primer; (**c**) attaching the CFRP plate with resin; (**d**) applying the resin to a part of the CFRP plate; (**e**) attaching the CFRP sheet; (**f**) the resulting strengthened RC beams.

Figure 8 illustrates the step-by-step procedure for installing CFRP to strengthen RC beams. It begins with turning the RC beam upside down, followed by surface preparation through roughening and cleaning, primer application, the attachment of CFRP plates with resin, the application of resin onto the CFRP plate surface, and the attachment of CFRP sheets.

The adhesive used for bonding CFRP plates and sheets was a two-part epoxy resin mixed at a 1:1 ratio (resin to hardener by weight). According to the manufacturer’s data, the epoxy exhibited a compressive strength of 78.72 MPa, a tensile strength of 54.10 MPa, and a flexural strength of 97.23 MPa. The bonding procedure was followed by curing at room temperature (20–25 °C) for 7 days to ensure full adhesion before testing.

### 2.6. Test Setup and Instrumentation

**Figure 9:**
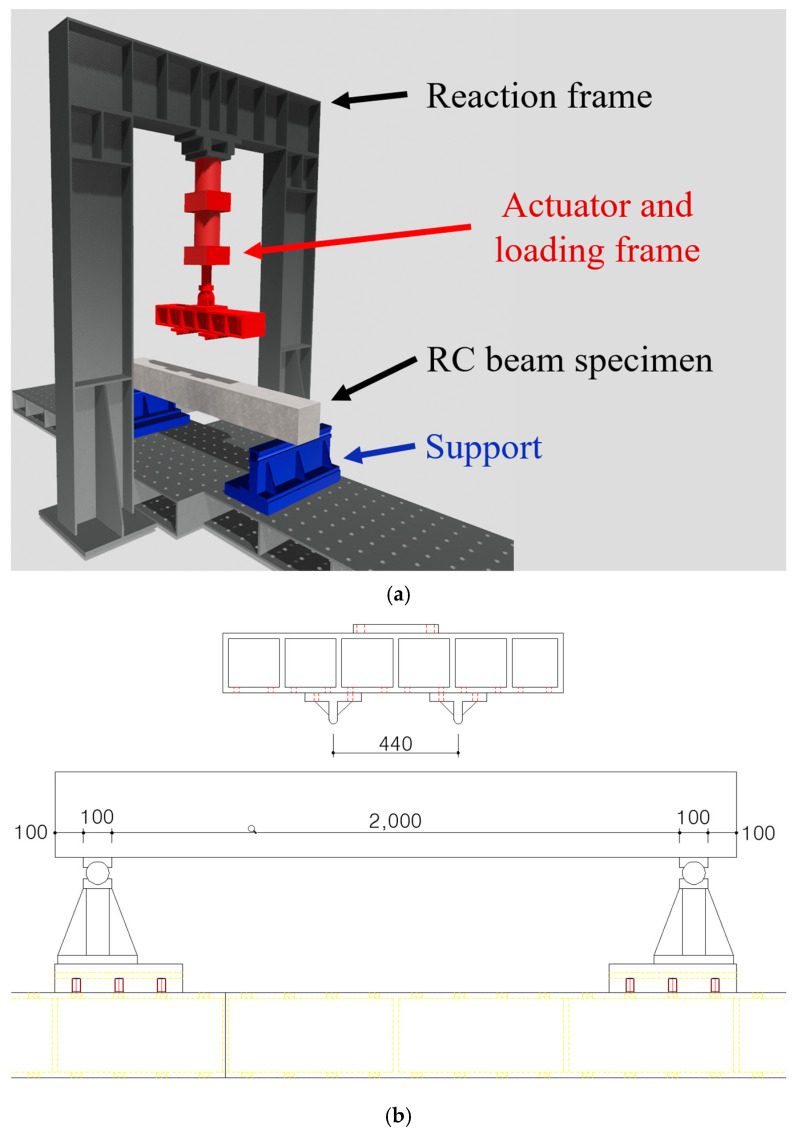
Test setup. (**a**) Schematic view. (**b**) Installation detail (unit: mm).

Each RC beam was tested under a symmetrical four-point bending test, as shown in Figure 9a. An electro-hydraulic actuator with a capacity of 500 kN was used for the four-point bending test. The four-point load details and locations are illustrated in Figure 9b. The distance between the two loading points was 400 mm, and the distance between the load point and the center of support was 850 mm. Mid-span deflection due to loading was measured using a linear variable differential transformer (LVDT).

### 2.7. Loading Protocol

**Figure 10:**
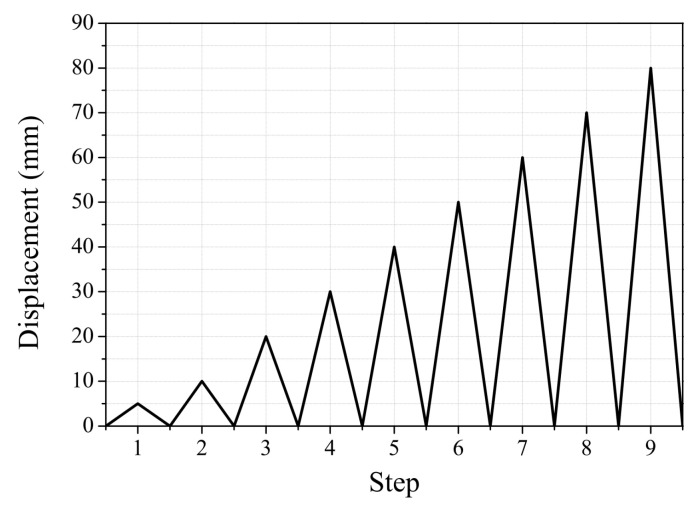
Loading protocol.

A cyclic loading protocol was used in the experiments. Cyclic testing with displacement control was conducted on all specimens at a speed of 5 mm per minute. The testing was performed in 5 mm increments from 0 to 10 mm and in 10 mm increments from 10 to 80 mm, as shown in Figure 10. Only positive cyclic loading was applied in this experiment to simulate the one-way bending condition typically observed in simply supported RC beams under gravity loads.

## 3. Test Results and Discussion

### 3.1. Failure Modes of RC Beams

**Figure 11:**
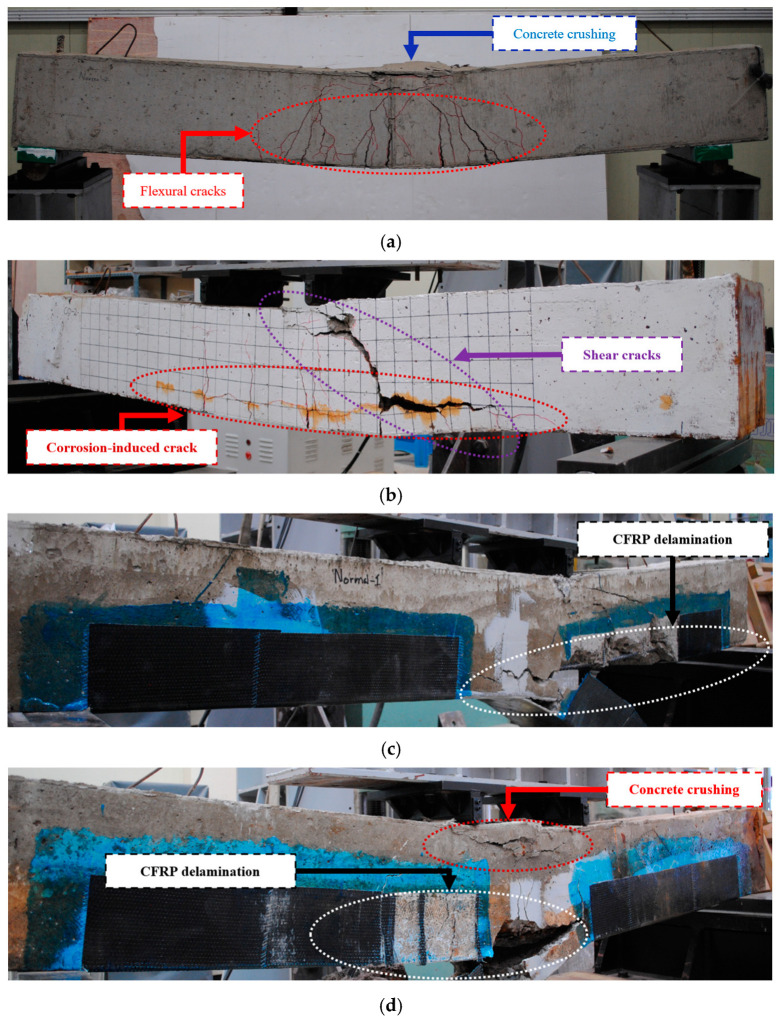
Final failure photographs of the tested reinforced concrete (RC) beams. (**a**) NB; (**b**) CB; (**c**) RNB; (**d**) RCB.

Figure 11 presents the failure modes of tested RC beams, including control specimens, corroded specimens, and those strengthened with CFRP plates and sheets. The reference specimen (NB) showed typical flexural failure, as depicted in Figure 10. Initially, flexural cracks emerged in the constant moment area, followed by their propagation upward into the compression zone and subsequently inclining toward the loading points. As the load increased beyond the yield point of the steel, deformation and crack width continued to increase. The loading was stopped when the concrete in the compression zone was crushed under compression.

In the damaged beam (CB), flexural cracks were initiated at lower loads than in the NB. This occurred due to the tensile stresses already induced by corrosion products on the concrete surrounding the rebars. The yielding of the longitudinal steel bars commenced at lower loads and displacements compared to the NB (refer to the following section), occurring at approximately 25% lower loads. The RC beam failure was initiated from the bottom as the stirrup corroded, following the yielding of the longitudinal steel bar. This resulted in the breaking of the stirrup and the evolution of flexural cracks toward the loading points, eventually leading to shear failure. The loading was stopped due to a sudden decrease in load caused by the widening of shear cracks.

The failure of the reinforced normal beam (RNB) and reinforced corroded beam (RCB) commenced with the appearance of flexural cracks. Subsequently, steel yielding and CFRP plate damage occurred, and the flexural crack originating from the point of CFRP plate failure evolved into a shear crack inclined toward the loading points. As the shear crack widened, the CFRP sheet tore, resulting in a Rip-Off Failure, where the CFRP plate detached from the underside concrete of the RC beam. RNB and RCB specimens exhibited CFRP plate rupture near the midspan rather than debonding at the ends, indicating that the U-wrap sheets provided sufficient anchorage. This failure mode confirms that the full capacity of the CFRP was activated, and premature debonding was effectively prevented.

### 3.2. Load History Curves of RC Beams

**Figure 12:**
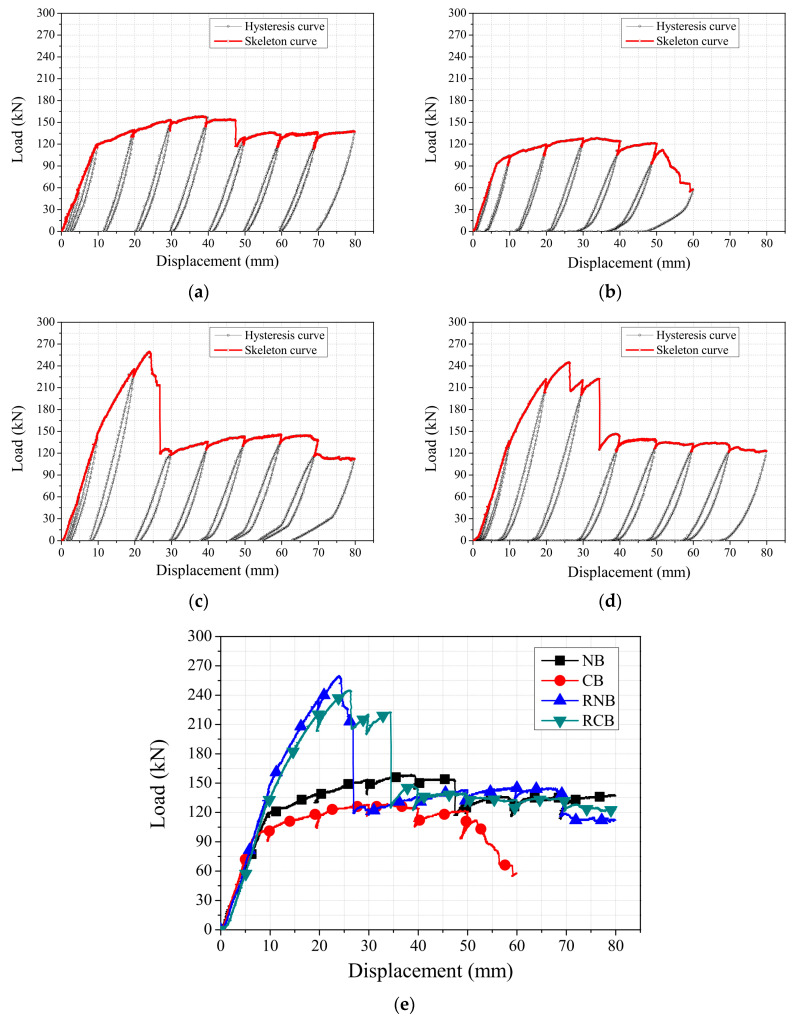
Hysteretic curve and skeleton curve of all tested reinforced concrete beams. (**a**) NB. (**b**) CB. (**c**) RNB. (**d**) RCB. (**e**) All.

The load–displacement curves for all the tested beams are shown in Figure 12a–d. In Figure 12e, the slopes of the curves for the corroded reinforced concrete beam (CB) were found to be smaller than that of the controlled beam (NB). In contrast, the RC beams strengthened with CFRP plates and sheets showed greater slopes in the load–displacement curves for the RNB compared to the RCB. This result indicates that the reduction in the slope of the curves due to the decrease in the cross-sectional area of the steel bars caused by corrosion is mitigated when strengthened with CFRPs. Consequently, increased stiffness occurs as the bending load is shared between the steel bars and the CFRP plates.

The yield and maximum strengths of the reinforced concrete beams, along with the corresponding displacements, are summarized in Table 3 based on the experimental results. For the NB and CB, the yield loads were 123.8 kN and 92.4 kN, and the peak loads were 162.5 kN and 129 kN, respectively. The RNB and RCB showed yield loads of 148 kN and 126.1 kN and peak loads of 260 kN and 245.1 kN, respectively. After experiencing a 10% weight loss in the steel bars due to corrosion, the yield load and peak load of the RC beam decreased by 25.4% and 20.6%, respectively, compared to the NB. This reduction is attributed to the loss of the cross-section of the corroded steel bars. Although the CB specimen showed slightly higher stiffness in the early loading stage compared to the NB, this is attributed to local surface cracking and slight loading eccentricity in the NB. Overall, the CB exhibited lower yield and peak load values, confirming the expected degradation due to corrosion. However, through strengthening with CFRP plates and sheets, the yield strength and peak strength of the NB increased by 19.5% and 60%, respectively, while those of the CB increased by 36.5% and 90%, respectively. This indicates that strengthening with CFRP plates and sheets is even more effective when applied after corrosion.

The ductility of each specimen was evaluated based on the ratio of ultimate displacement to yield displacement. As shown in Table 4, the calculated ductility indices were 9.09 for NB, 9.23 for CB, 7.92 for RNB, and 8.25 for RCB. Interestingly, the CB specimen, despite its corrosion-induced degradation, exhibited a slightly higher ductility than NB due to early yielding and extended deformation capacity under lower load levels. On the other hand, both CFRP-strengthened specimens (RNB and RCB) demonstrated slightly reduced ductility compared to their unstrengthened counterparts. This reduction is attributed to the brittle nature of CFRP, which enhances strength but limits post-yield deformation. Nevertheless, the ductility values of RNB and RCB remain within acceptable ranges, indicating that CFRP reinforcement improved load-carrying capacity without significantly compromising overall deformability.

### 3.3. Energy Dissipation

**Figure 13:**
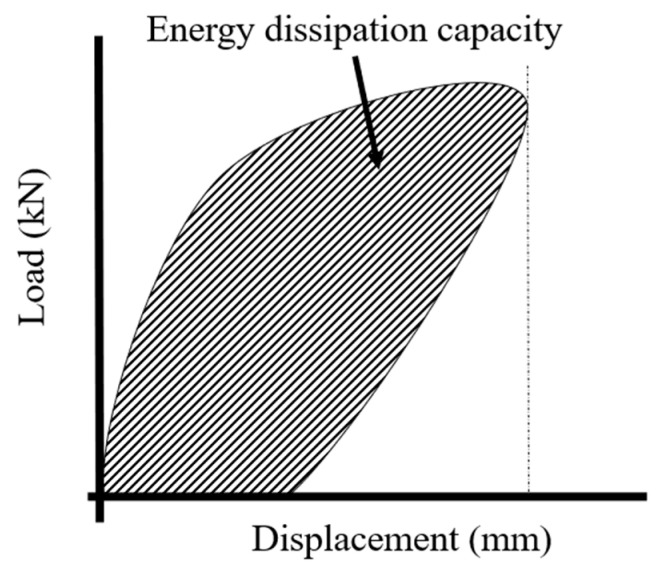
Determination of energy dissipation capacity.

Energy dissipation capacity refers to the ability to absorb external forces until structural failure. Since energy dissipation due to cyclic behavior represents a structure’s seismic performance, evaluating the energy dissipation capacity of the structure is crucial [34]. To assess energy dissipation capacity, this study designed cyclic testing with displacement control on the RC beams according to each target displacement (step). The energy dissipation capacity was calculated by the area enclosed by the load history curve for one cycle and bounded by the X and Y axes, as shown in Figure 13.

**Figure 14:**
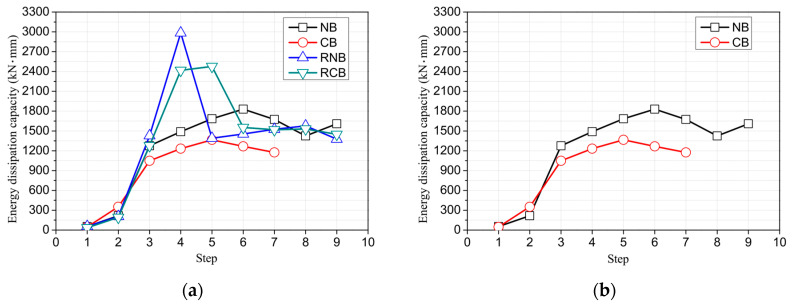

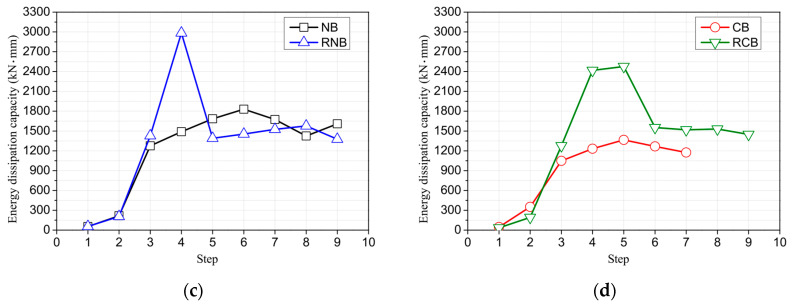
The energy dissipation capacity of tested reinforced concrete beams. (**a**) All. (**b**) Comparison of corroded specimens. (**c**) Comparison of CFRP strengthening. (**d**) Comparison of CFRP strengthening after corrosion.

**Figure 15:**
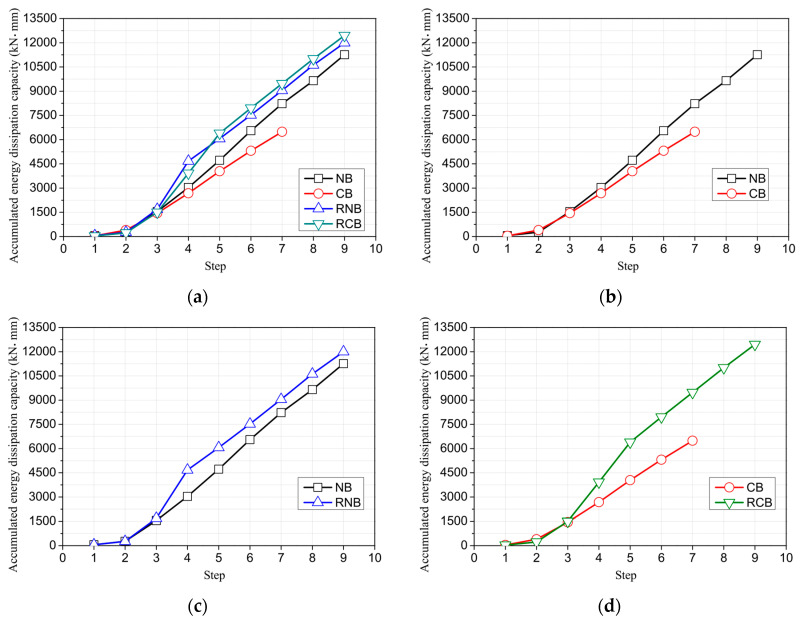
Cumulated energy dissipation capacity of tested reinforced concrete beams. (**a**) All. (**b**) Comparison of corroded specimens. (**c**) Comparison of CFRP strengthening. (**d**) Comparison of CFRP strengthening after corrosion.

Figure 14a presents the energy dissipation capacity of all the tested RC beams. As seen in Figure 14b, corrosion reduces the energy dissipation capacity, but, as shown in Figure 14c,d, CFRP reinforcement can enhance it. Figure 15 displays the accumulated energy dissipation capacity, which is the sum of energy dissipation due to continuous cyclic loading during experiments, reflecting the total energy lost due to inelastic deformation. Corrosion damage reduces the accumulated energy dissipation capacity of the NB by 42%. However, strengthening the corroded RC beam (CB) with CFRP plates and sheets results in a 92% increase in the accumulated energy dissipation capacity compared to the CB. This increase far surpasses the 7% increase in accumulated energy dissipation capacity observed when strengthening the NB with CFRP plates and sheets. It suggests that the CFRP strengthening technique devised in this study effectively enhances the accumulated energy dissipation capacity of RC beams damaged by corrosion. However, the reinforcing effect on the accumulated energy dissipation capacity of controlled RC beams, which remain undamaged, is almost negligible. The energy dissipation capacity (EDC) and accumulated energy dissipation capacity (AEDC) at each cyclic loading displacement are summarized in Table 5.

## 4. Conclusions

To investigate the structural performance of RC beams reinforced with CFRP plates and CFRP sheets under conditions of corrosion, this study conducted experiments on four RC beam specimens subjected to cyclic loading. The experimental results considered various structural performance aspects of RC beams, including failure modes, load–displacement relationships, and energy dissipation capacity.

The analysis of the failure modes of the tested RC beams reveals that the NB exhibited typical flexural failure, with concrete crushing under compression. In contrast, the CB exhibited flexural cracks at lower loads compared to the NB. Following rebar yielding, the corrosion of the stirrups initiated RC beam failure from the bottom, resulting in stirrup fracturing and the propagation of flexural cracks toward the loading points, ultimately leading to shear failure. Conversely, the RNB and the RCB progressed from flexural to shear failure, resulting in a Rip-Off Failure whereby the CFRP plate detached from the underside concrete of the RC beam.

The analysis of load–displacement curves for all tested beams revealed that the slope of the curves for the CB was smaller than that of the NB. However, RC beams strengthened with CFRP exhibited a greater slope compared to the NB. This suggests that CFRP reinforcement mitigates the decrease in slope caused by a corrosion-induced reduction in the rebar cross-sectional area, resulting in increased stiffness as the load is distributed between CFRP plates and rebars during bending. Additionally, reinforcing with CFRP increased the yield and peak loads of both the NB and the CB, with the increase being more pronounced in the CB. This highlights the effectiveness of CFRP reinforcement, especially when applied post-corrosion.

The analysis of energy dissipation capacity for all tested beams revealed that corrosion reduces energy dissipation capacity, but it can be enhanced through CFRP reinforcement. Corrosion damage resulted in a 42% decrease in the accumulated energy dissipation capacity of the NB, while reinforcing corroded RC beams (CBs) with CFRP plates and CFRP sheets led to a 92% increase in accumulated energy dissipation capacity compared to the CB. This increase is significantly higher than the observed 7% increase when reinforcing the NB with CFRP plates and sheets.

## Figures and Tables

**Table 1:** The design parameters of the specimens.

Specimen Label	Corrosion Ration	Strengthening Method
NB	0%	Unstrengthened
CB	10%	Unstrengthened
RNB	0%	Strengthened with CFRP plate + CFRP sheet
RCB	10%	Strengthened with CFRP plate + CFRP sheet

**Table 2:** Material properties of carbon fiber-reinforced polymer (CFRP) reinforcements.

	Strength at Max. Load (MPa)	Strain at Max. Load	Modulus of Elasticity (GPa)	Thickness (mm)	Width (mm)
CFRP plate	621.34	0.024	25.89	0.5	25
CFRP sheet	1367.72	0.010	136.77	0.4	25

**Table 3:** Test results of the reinforced concrete beams.

Specimen Labels	Yield Load (kN)	Disp. at Yield Load (mm)	Peak Load (kN)	Disp. at Peak Load (mm)
NB	123.8	8.8	162.5	37.5
CB	92.4	6.5	129.0	33.7
RNB	148.0	10.1	260.0	23.9
RCB	126.1	9.7	245.1	26.1

**Table 4:** Ductility index of the reinforced concrete beams.

Specimen Labels	Yield Disp. (mm)	Ultimate Disp. (mm)	Ductility Index (Ultimate Disp. /Yield Disp.)
NB	8.8	80	9.09
CB	6.5	60	9.23
RNB	10.1	80	7.92
RCB	9.7	80	8.25

**Table 5:** Energy dissipation capacity of all tested reinforced concrete (RC) beams.

Specimen Labels		Step								
		1	2	3	4	5	6	7	8	9
NB	EDC ^1^	54	216	1276	1488	1685	1830	1674	1424	1608
	AEDC ^2^	54	270	1546	3035	4721	6551	8226	9650	11,259
CB	EDC	46	352	1048	1232	1365	1267	1175		
	AEDC	46	398	1446	2678	4043	5310	6485		
RNB	EDC	56	207	1428	2984	1390	1455	1525	1578	1377
	AEDC	56	263	1691	4675	6065	7520	9045	10,623	12,000
RCB	EDC	35	193	1278	2417	2476	1553	1519	1531	1449
	AEDC	35	228	1506	3923	6399	7952	9471	11,002	12,451

^1^ EDC: energy dissipation capacity, ^2^ AEDC: accumulated energy dissipation capacity.

## Data Availability

The original contributions presented in this study are included in the article. Further inquiries can be directed to the corresponding authors.
